# Application of the Denitrification-Decomposition Model to Predict Carbon Dioxide Emissions under Alternative Straw Retention Methods

**DOI:** 10.1155/2013/851901

**Published:** 2013-12-25

**Authors:** Can Chen, Deli Chen, Jianjun Pan, Shu Kee Lam

**Affiliations:** ^1^College of Resources and Environmental Sciences, Nanjing Agricultural University, Nanjing, Jiangsu 210095, China; ^2^Melbourne School of Land and Environment, The University of Melbourne, Melbourne, VIC 3010, Australia

## Abstract

Straw retention has been shown to reduce carbon dioxide (CO_2_) emission from agricultural soils. But it remains a big challenge for models to effectively predict CO_2_ emission fluxes under different straw retention methods. We used maize season data in the Griffith region, Australia, to test whether the denitrification-decomposition (DNDC) model could simulate annual CO_2_ emission. We also identified driving factors of CO_2_ emission by correlation analysis and path analysis. We show that the DNDC model was able to simulate CO_2_ emission under alternative straw retention scenarios. The correlation coefficients between simulated and observed daily values for treatments of straw burn and straw incorporation were 0.74 and 0.82, respectively, in the straw retention period and 0.72 and 0.83, respectively, in the crop growth period. The results also show that simulated values of annual CO_2_ emission for straw burn and straw incorporation were 3.45 t C ha^−1^ y^−1^ and 2.13 t C ha^−1^ y^−1^, respectively. In addition the DNDC model was found to be more suitable in simulating CO_2_ mission fluxes under straw incorporation. Finally the standard multiple regression describing the relationship between CO_2_ emissions and factors found that soil mean temperature (SMT), daily mean temperature (*T*_mean_), and water-filled pore space (WFPS) were significant.

## 1. Introduction

Atmospheric CO_2_ concentrations have increased by approximately 35% and are predicted to reach 700 ppmv by the end of 20 century [1]. Soils are the largest carbon pool in terrestrial ecosystem, containing more than two thirds of the total carbon and soil respiration, and contribute an annual atmospheric CO_2_ flux 10 times greater than fossil fuel combustion [2]. Therefore, it is crucial to reduce CO_2_ emissions from agricultural land.

There are many factors affecting CO_2_ emissions, including soil temperature, soil moisture, and soil organic matter (SOM) [3]. Schlesinger and Andrews [4] showed that soil CO_2_ emissions increase with soil temperature. La Scala et al. [5] reported that microbial activity, soil respiration, and enzymatic activity increased with rising temperature. Subke et al. [6] suggested that soil moisture was an important nonbiological factor affecting soil CO_2_ flux. Li [7] analyzed the sensitivity in predicting CO_2_ and N_2_O flux emissions for a series of models and found that the amount of soil organic matter (SOM) was the most important factor.

Straw retention has been adopted worldwide to increase crop production. It has been shown to reduce CO_2_ but increase crop yield [8]. Li et al. [9] considered that if the straw retention rate increased from 15 to 80% in China, the Chinese agricultural carbon budget should change from negative (emissions from soil of −9.5 × 10^7^ t y^−1^) to positive (soil absorbing +8.0 × 10^7^ t y^−1^). While the effects of different straw retention methods on CO_2_ emission flux have been studied in many continuous long-term experiments, information on the use of the DNDC model to simulate CO_2_ emission under different straw retention scenarios is lack. In particular information is required to predict the total variation of CO_2_ emission fluxes and the interrelation of important factors. The objective of this study was to simulate the daily rate of CO_2_ emissions in the straw retention and crop growth periods and study the annual amount of CO_2_ emission by the DNDC model for different straw retention treatments. The research analyzed the relationship of the major factors with path analysis and provided implications for the mitigation of CO_2_ emissions in the study area.

## 2. Materials and Methods

### 2.1. Study Site

Field experiments were conducted on a commercial farm in New South Wales (NSW) Australia, 34°30′S, 146°11′E, located approximately 30 km southeast of Griffith. Mean annual precipitation is 432 mm, and mean maximum and minimum temperatures are 23.0 and 10.3°C, respectively (measured at the nearest recording station, Leeton). The soil (0–20 cm) is classified as a Typic Natrixeralf and Mundiwa clay loam with clay particle content in 53.11% [10]. The surface soil has a pH of 5.5 (soil : water = 1 : 5) of 0.03 kg carbon (C)/kg and soil bulk density of 1.37 g cm^−3^ (Table 1). WFPS is calculated as WFPS = (soil gravimetric water content × bulk density)/(1 − (bulk density/particle density)) [10]. Maize was grown at the site on beds (two rows of plants per bed) and irrigated by furrow irrigation.

### 2.2. Experimental Design and Data Analysis

The field experiment began on 11 May 2010 (day 1) and ended on 10 May 2011 (day 365). There were two maize straw treatments in the field experiment. A randomized block design with three replicates was used in the 12 plots. The maize straw treatments were (1) application of 300 kg N ha^−1^, maize straw burnt and left on the field (300N-burn), and (2) application of 300 kg N ha^−1^, maize straw mulched (the amount of maize straw was 6750 kg ha^−1^) and incorporated into soil (5 cm) soon after harvest (300N-incorporated). The 300N-burn treatment used 6 plots and 300N-incorporated treatment used another 6 plots. The result of each treatment was the mean value. The two straw retention methods lasted for one maize season. Fertilizer was applied three times: 90 kg N ha^−1^ as (NH_4_)_2_  HPO_4_ on October 22, 2010 (day 165) three days before sowing, 10 kg N ha^−1^ as urea (mixed with the soil as seed manure) on October 25, 2010 (day 168) at sowing, and 200 kg N ha^−1^ as urea on December 6, 2010 (day 204). The area was flood irrigated ten times (Table 2). This study was divided into two periods. The first period, from May 11, 2010 to October 22, 2010 was called straw retention period. The second period, from October 22, 2010 to May 10, 2011 was called maize growth period.

The CO_2_ fluxes from the soil-plant system were measured on the basis of static transparent chamber and gas chromatography methods [3]. The bottom chambers were empty and the top chambers were connected to an infrared CO_2_ analyzer via air pipes. Chambers were 43 cm × 43 cm × 110 cm and were adjusted according to the height of maize across time [11]. This system measures fluxes integrated over 2 or 3 day intervals and avoids errors associated with manual flux chambers, when measurements are taken only at a particular time period. Each system was powered by a 12 V 120 amp-hour battery. The battery capacity was supplemented by an 80 W solar panel that extended the period between battery changeover to between 1 and 4 weeks. Soil temperature was measured using type K thermocouples and soil moisture was measured using time domain reflectometry (TDR) (Theta Probes ML2x, Delta-T Devices Ltd., Cambridge, UK). Both sets of sensors were logged by a controller/logger unit. Gas concentrations in the Tedlar sample bags were measured off-site at Aspendale. CO_2_ was measured using a Licor 6251 NDIR (Licor, Nebraska, USA). In addition CO_2_ concentration was measured directly in the field using a Gascard_II CO_2_ sensor (Edinburgh Instruments, Edinburgh, UK) incorporated into each chamber controller.

### 2.3. DNDC Model

In this study the DNDC model (version 9.5; http://www.dndc.sr.unh.edu/) was applied to simulate CO_2_ emission under different straw retention scenarios. The DNDC model has a relatively high level of complexity. The DNDC model can be used to simulate fluxes of CO_2_, H_2_O, N_2_O, N_2_, CH_4_, leaf area index (LAI) development, soil organic matter decomposition rate, nutrient leaching, change in soil organic carbon (SOC), and biomass production [12]. DNDC contains four main submodels [7, 13]. The soil climate sub-model calculates hourly and daily soil temperature and moisture fluxes. The crop growth submodel simulates crop biomass accumulation and partitioning. The decomposition sub-model calculates decomposition, nitrification, NH_3_ volatilization and CO_2_ production. The input data are shown in Tables 1 and 2.

### 2.4. Data Analysis

The DNDC model was used to simulate CO_2_ fluxes under the different straw retention methods. Model accuracy and performance were evaluated by calculating the correlation coefficient and model efficiency (ME) [14]. ME is calculated as

(1) $$\begin{array}{c} \text{ME}=1-\;\frac{\sum_{i=1}^{n}{\left(P-O\right)}^{2}}{\sum_{i=1}^{n}{\left(O_{i}-\overset{-}{O}\right)}^{2}}, \end{array}$$

where *O* is observed values, *P* is simulated values, *n* is the total number of observations, $\overset{-}{O}$ is the mean of observed values, and *i* is the current observation.

ME compares the squared sum of the absolute error with the squared sum of the difference between the observations and their mean value. It compares the ability of the model to reproduce the daily data variability with a much simpler model that is based on the arithmetic mean of the measurements. ME values close to 1 indicate a “near-perfect” fit [15, 16].

Five continuous long-term measurement factors were considered for the statistical analysis, namely, daily maximum temperature (*T*_max⁡_), daily minimum temperature, (*T*_min⁡_), daily mean temperature (*T*_mean_), soil mean temperature (SMT), and amount of irrigation. Irrigation was not a daily operation, but it had residual effects on soil water. Therefore, water-filled pore space (WFPS) was used to reflect the water dynamics following irrigation and rainfall events in soil. As a result, five factors which affected the CO_2_ emission were selected: *T*_max⁡_, *T*_min⁡_, *T*_mean_, SMT, and WFPS.

Data were analyzed by correlation analysis and path analysis using SPSS 13.0. Path analysis can be used for the analysis of multiple variables and the linear relationship between variables. It was a development of regression analysis [17].

## 3. Results

The straw retention period and crop growth period were studied separately because the sources of the CO_2_ were different in these two periods [18]. The main sources of CO_2_ were straw decomposition, SOM decomposition, and microbial respiration during the straw retention period. Root respiration, SOM decomposition, and microbial respiration are the main sources during the crop growth period [19–21].

### 3.1. Simulation of Daily CO_2_ Emission during Straw Retention Period

**Figure 1:**
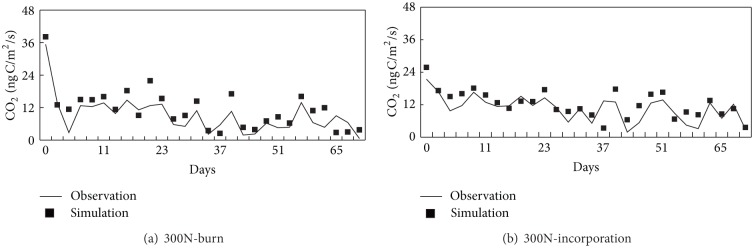
Comparison of observed and simulated CO_2_ flux during straw retention period.

The simulated and observed values of daily CO_2_ emission under different straw retention treatments during straw retention period are shown in Figure 1. The correlation coefficient between simulated and observed values for treatments 300N-burn and 300N-incorporation were 0.74 (Figure 1(a)) and 0.82 (Figure 1(b)), respectively, (*n* = 74) and the ME values were 0.63 and 0.76.

### 3.2. Daily CO_2_ Emission during Crop Growth and Annual CO_2_ Emissions

**Figure 2:**
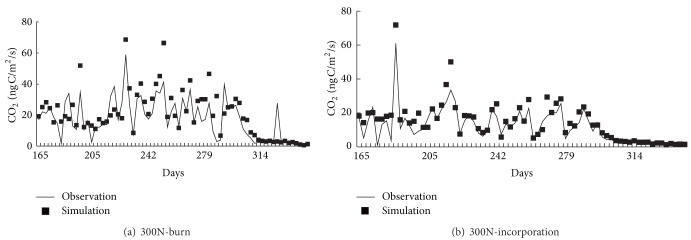
Comparison of observed and simulated CO_2_ flux during crop growth period.

The DNDC model was also used to simulate the daily CO_2_ fluxes during crop growth (Figure 2).

The correlation coefficients between the observed and simulated values of CO_2_ flux were 0.72 and 0.82 (*n* = 186) for treatment 300N-burn and 300N-incorporation, respectively (Figure 2). The corresponding ME values were 0.63 and 0.79. The ME value of 300N-incorporation was higher than that of the 300N-burn. This indicates that the DNDC model was more suitable for simulating CO_2_ fluxes for the straw incorporation treatment than the straw burning treatment during the crop growth period.

The observed values of CO_2_ emissions during the maize growth season for 300N-Burn and 300N-incorporation were 4.7 t C ha^−1^ y^−1^ and 3.5 t C ha^−1^ y^−1^, respectively. The corresponding simulated values were 3.45 t C ha^−1^ y^−1^ and 2.13 t C ha^−1^ y^−1^, respectively (Table 3). The observed values were smaller than the observed values. This is because DNDC model could simulate the CO_2_ which is discharged by the microbial activities. The CO_2_ which produced though plant root respiration was ignored. This also suggested that the DNDC model might have underestimated the microbial activity and the rate of SOM decomposition [3]. So it is necessary to improve the DNDC model to adjust microbial activity and the rate of SOM decomposition simulation and contain the plant root respiration simulation function.

### 3.3. Sensitive Analysis

Fixed factors (continuous long-term measurements) were used for the sensitivity analysis. Because the DNDC model was more suitable for the simulation of CO_2_ emissions under straw incorporation than the burning of straw, the reason is shown in Sections 3.1 and 3.2, the sensitivity analysis mainly focused on the relationship between different factors and CO_2_ emission for the straw incorporation treatment.

The CO_2_ emissions were significantly correlated with *T*_max⁡_, *T*_min⁡_, *T*_mean_, WFPS, and SMT. The correlation coefficients were 0.5681, 0.5114, 0.5125, 0.5366, and 0.6729, respectively (*P* < 0.01) (Table 4). Temperature, WFPS, and microbial activity can influence SOM decomposition and associated CO_2_ emissions [5, 6, 19].

Path analysis was used to analyze the relationship among these five factors (Tables 5 and 6). The results showed that SMT, *T*_mean_, and WFPS were the main controlling factors of CO_2_ emissions. The standard multiple regression equation of the CO_2_ emission flux was *Y* = −34.113 + 0.8067*X*_1_ + 0.6392*X*_2_ + 0.4014*X*_3_ (*r* = 0.964, *P* < 0.01, *n* = 260), where *Y* is CO_2_ emission fluxes, *X*_1_ is SMT, *X*_2_ is *T*_mean_, and *X*_3_ is WFPS. The *T*_max⁡_ and *T*_min⁡_ were not chosen because of large diurnal fluctuation of temperature in the Griffith region. The daily maximum temperature and daily minimum temperature did not reach the optimum temperature for microbial activity.

## 4. Discussion

### 4.1. The discussion of Daily CO_2_ Emission during Straw Retention Period

The observed and simulated CO_2_ emission decreased with time regardless of straw retention treatment (Figure 1). This was because the main source of CO_2_ emission was straw decomposition in the straw retention period [20]. The straw decomposition rate decreased with time. The simulated and observed CO_2_ fluxes in the straw burnt treatment were higher than those under straw incorporation. The reason for the difference may be related to soil structural differences, particularly the reduced accessibility of N by plant roots in the burned treatment [22]. This difference may have contributed to the greater N_2_O emissions from soil in our straw burn treatments. Perhaps the process was the same in the CO_2_ experiments. The change in soil structural in the straw burn treatment may allow microbes to obtain more mineral nutrition after straw burn and promote the formation of granular structure. Ruser et al. [23] investigated the impact of compaction on soils from a row cropping system and found that CO_2_ production has a sensitive influence on the soil compaction. The other reason is in agreement with the finding by Beer et al. [24] that more greenhouse gas was evolved from straw burn treatments monitored using automatic chambers over the whole season. Therefore the straw burn treatment discharged more CO_2_ than the straw incorporation treatment, so that the CO_2_ emissions were lower in the straw incorporation than in the straw burn [9].

The correlation coefficient between simulated and observed values and ME values implies that the DNDC model can be used to simulate daily CO_2_ fluxes in the straw retention period. The ME value for 300N-incorporation was higher than for 300N-burn, suggesting that the DNDC model was more suitable to simulate CO_2_ emission under straw incorporation than under the burning of straw.

### 4.2. The Discussion of Daily CO_2_ Emission during Crop Growth and Annual CO_2_ Emissions

Both the observed and simulated values for the treatment 300N-burn were higher than those for the 300N-incorporation. The result was the same as the straw retention method. Straw decomposition rate varies with the depth of incorporation [25]. It has been shown that the straw decomposition rate during the 32 weeks of study was the highest at the 5 cm soil depth (decomposed > 65%), followed by the 15 cm soil depth (62%), the lowest for the straw materials left on the soil surface (50%) [7]. Under the 300N-burn treatment, the maize straw was burnt and left on the field, and the main products of maize straw combustion were CO_2_ and plant ash. CO_2_ was emitted to the atmosphere directly. Plant ash was the main driver of microbial activity. Part of the plant ash was used by microbes in the straw retention period and the other part was used in the crop growth period. So increasing soil organic carbon associated with straw incorporation [26] would drive decreased CO_2_ emission. On the other hand, the base of plant ash contains substantial mineral nutrients, which could be used by the microbes directly for energy [27, 28]. Wakelin et al. [29] found that stubble burnt and incorporation led to totally dissimilar soil microbial populations. The straw, which was incorporated into the soil, might require a long organic matter decomposition process [7, 30]. The soil organic matter decomposition process is slow. Therefore, the soil microbes in the straw incorporated treatment might obtain less energy than in the soil burn treatment, so that straw incorporation discharges CO_2_ slower than straw burn.

The correlation coefficient between simulated and observed values and ME values indicates that the DNDC model was more suitable for simulating CO_2_ fluxes for the straw incorporation treatment than the straw burning treatment during the crop growth period.

### 4.3. The Discussion of Sensitive Analysis

SMT, *T*_mean_, and WFPS showed a direct positive effect (the corresponding direct path coefficients were 0.8067, 0.6392, and 0.4014, resp.) and indirect positive effect (the total indirect path coefficients were 1.1016, 1.2499, and 1.0915, resp.) on CO_2_ emissions (Table 6). This indicates that SMT, *T*_mean_, and WFPS could directly and indirectly affect the microbial activity and decomposition of straw and organic matter in soil and control CO_2_ emissions. The total indirect path coefficients of SMT, *T*_mean_, and WFPS were greater than direct path coefficients. SMT, *T*_mean_, and WFPS could influence the microbes in decomposing the organic matter and straw to release CO_2_ [9]. The WFPS included irrigation and rainfall events in soil. So irrigation and rainfall events into soil may mainly indirectly affect CO_2_ emissions.

## 5. Conclusions

The DNDC model can be used to simulate CO_2_ emissions under different straw retention practices in the Griffith region, NSW, Australia. The results showed that the simulation values and trends were very close to the measured values of daily CO_2_ fluxes, CO_2_ annual emissions, and emission factors for all straw retention methods. The model accuracy for the 300N-incorporated treatment was higher than that for the 300N-burn treatment in both the straw retention period and the crop growth period. This implies that the DNDC model is more appropriate for simulation of CO_2_ emissions under straw incorporation treatment.

CO_2_ emissions were positively correlated with *T*_max⁡_, *T*_min⁡_, *T*_mean_, WFPS, and SMT. The path analysis showed the standard multiple regression equation of the CO_2_ emission was *Y* = −34.113 + 0.8067*X*_1_ + 0.6392*X*_2_ + 0.4014*X*_3_. The SMT, *T*_mean_, and WFPS were the main factors influencing CO_2_ emission under different straw retention methods. Management of these practices will help mitigate CO_2_ emissions in cropping systems

## Figures and Tables

**Table 1:** Input parameters used in the DNDC model (0–20 cm).

Parameter	Soil bulk density (g cm^−3^)	pH	Field capacity (%)	Wilting point (%)	Clay fraction (%)	SOC in the surface soil (kg C kg^−1^)	C/N	Initial NO_3_^−^ (mg N kg^−1^)	Initial NH_4_^+^ (mg N kg^−1^)
Data	1.37	5.5	38.01	10.22	53.11	0.03	10.90	6.30	3.32

**Table 2:** Irrigation times and amount of water used in each irrigation.

Data	Irrigation amount (mm)	Data	Irrigation amount (mm)
28/10/2010	197	26/11/2010	200
18/12/2010	127	27/12/2010	90
5/1/2011	82	13/1/2011	102
28/1/2011	61	15/2/2011	74
24/2/2011	76	3/3/2011	76

**Table 3:** The observed and simulated annual CO_2_ emission for the maize season.

	300N-burn (t C ha^−1^ year^−1^)	300N-incorporation (t C ha^−1^ year^−1^)
CO_2_-observed values	4.7	3.5
CO_2_-simulated values	3.45	2.13

**Table 4:** Correlation coefficients between CO_2_ and same soil variables.

	*T*_max⁡_	*T*_min⁡_	*T*_mean_	WFPS	SMT	CO_2_
*T*_max⁡_	1.0000	0.7967**	0.9592**	0.5525**	0.7259**	0.5681**
*T*_min⁡_		1.0000	0.9350**	0.6952**	0.7123**	0.5114*
*T*_mean_			1.0000	0.6494**	0.912**	0.5125**
WFPS				1.0000	0.6307**	0.5366**
SMT					1.0000	0.6729**
CO_2_						1.0000

**Correlation is significant at the 0.01 level-2-tailed).*Correlation is significant at the 0.05 level-2-tailed).

**Table 5:** The standard multiple regression coefficients.

Unstandardized coefficients				Standardized coefficients		
Model		*B*	Std.error	Beta	*t*	Sig.
1	(Constant)	−3.832	2.089	—	1.835	0.069
	Stemper	0.316	0.018	0.573	7.430	0.000
⋮	⋮	⋮	⋮	⋮	⋮	⋮
3	(Constant)	−34.113	7.434	—	4.098	0.000
	SMT	0.8067	0.030	0.452	3.360	0.000
	*T*_mean_	0.6392	0.152	0.681	2.135	0.000
	WFPS	0.4014	0.237	0.339	2.672	0.005

**Table 6:** Path coefficient of each factor on CO_2_ emission.

Soil factor	Direct path coefficient	Indirect path coefficient				
		*X*_1_ (SMT)	*X*_2_ (*T*_mean_)	*X*_3_ (WFPS)	Total	Error path coefficient
*X*_1_ (SMT)	0.8067	1	0.5830	0.2531	1.1016	0.2363
*X*_2_ (*T*_mean_)	0.6392	0.7357	1	0.2607	1.2499
*X*_3_ (WFPS)	0.4014	0.5088	0.4151	1	1.0915
